# Mapping Factors That Affect the Uptake of Digital Therapeutics Within Health Systems: Scoping Review

**DOI:** 10.2196/48000

**Published:** 2023-07-25

**Authors:** Robin van Kessel, Andres Roman-Urrestarazu, Michael Anderson, Ilias Kyriopoulos, Samantha Field, Giovanni Monti, Shelby D Reed, Milena Pavlova, George Wharton, Elias Mossialos

**Affiliations:** 1 LSE Health Department of Health Policy London School of Economics and Political Science London United Kingdom; 2 Department of International Health Care and Public Health Research Institute, Faculty of Health, Medicine and Life Sciences Maastricht University Maastricht Netherlands; 3 Department of Psychiatry University of Cambridge Cambridge United Kingdom; 4 Department of Psychiatry and Behavioral Sciences Stanford University Stanford, CA United States; 5 Department of Health Services Research and Policy London School of Hygiene and Tropical Medicine London United Kingdom; 6 Duke Clinical Research Institute Duke University School of Medicine Durham, NC United States; 7 Department of Health Services Research Care and Public Health Research Institute, Faculty of Health Medicine and Life Science Maastricht University Maastricht Netherlands; 8 Institute of Global Health Innovation Imperial College London London United Kingdom

**Keywords:** digital health, uptake, implementation, adoption, framework, digital therapeutics, scoping review, thematic analysis, digital medicine, policy

## Abstract

**Background:**

Digital therapeutics are patient-facing digital health interventions that can significantly alter the health care landscape. Despite digital therapeutics being used to successfully treat a range of conditions, their uptake in health systems remains limited. Understanding the full spectrum of uptake factors is essential to identify ways in which policy makers and providers can facilitate the adoption of effective digital therapeutics within a health system, as well as the steps developers can take to assist in the deployment of products.

**Objective:**

In this review, we aimed to map the most frequently discussed factors that determine the integration of digital therapeutics into health systems and practical use of digital therapeutics by patients and professionals.

**Methods:**

A scoping review was conducted in MEDLINE, Web of Science, Cochrane Database of Systematic Reviews, and Google Scholar. Relevant data were extracted and synthesized using a thematic analysis.

**Results:**

We identified 35,541 academic and 221 gray literature reports, with 244 (0.69%) included in the review, covering 35 countries. Overall, 85 factors that can impact the uptake of digital therapeutics were extracted and pooled into 5 categories: policy and system, patient characteristics, properties of digital therapeutics, characteristics of health professionals, and outcomes. The need for a regulatory framework for digital therapeutics was the most stated factor at the policy level. Demographic characteristics formed the most iterated patient-related factor, whereas digital literacy was considered the most important factor for health professionals. Among the properties of digital therapeutics, their interoperability across the broader health system was most emphasized. Finally, the ability to expand access to health care was the most frequently stated outcome measure.

**Conclusions:**

The map of factors developed in this review offers a multistakeholder approach to recognizing the uptake factors of digital therapeutics in the health care pathway and provides an analytical tool for policy makers to assess their health system’s readiness for digital therapeutics.

## Introduction

### Background

The digital health landscape encompasses a broad range of technologies that promote, improve, or support health system functioning and the delivery of health care, including electronic health records, telemedicine, mobile health apps, and health data analytics [1]. Digital therapeutics are a specific subset of the overarching digital health landscape that generate and deliver clinically validated medical interventions and are used as part of a clinical treatment pathway for various health conditions [2,3]. As a result, they can be regulated and prescribed as therapeutics or medical devices, which distinguishes them from more generic digital health applications in the well-being and lifestyle space [4].

Despite digital therapeutics being used to successfully treat a range of conditions, including insomnia, attention-deficit/hyperactivity disorder in children, and substance use disorder [5-9], as well as showing cost-reducing properties [8,10], there are several barriers that reduce their uptake. These include issues around reimbursing them [11,12] and a lack of standardization in evaluating the technologies and their outcome measures. This makes it difficult to use standard approaches to compare different interventions using traditional clinical and cost-effectiveness evaluations [1,11,13,14]. The resulting lack of data on the comparative effectiveness and cost-effectiveness of digital therapeutics can limit market access for innovative technologies [13,15] and present challenges for policy makers and providers when establishing reimbursement mechanisms. There are also factors that influence uptake at the patient level, including digital infrastructure and literacy [2,15-18], and at the health professional level, including lack of training, uncertainties surrounding accountability, and shifts in professional workflow [13,19]. Although the COVID-19 pandemic boosted the implementation of digital health across the health ecosystem [17,18,20], the uptake was not sustainable, as patients reverted back to traditional health services over time [21].

### Objective

A number of studies have identified a range of barriers to the uptake of digital health products at different levels [15,19,22,23]. Understanding the full spectrum of uptake factors is essential to identify ways in which policy makers and providers can facilitate the adoption of effective digital therapeutics within a health system, as well as the steps developers can take to assist in the deployment of products [24,25]. In this review, we aimed to map the most frequently discussed uptake factors of digital therapeutics using a scoping review of the digital health literature.

## Methods

### Overview

We performed a scoping review with thematic synthesis in accordance with the scoping review framework developed by Arksey and O’Malley [26] and Levac [27]. This method allows for the rapid mapping of the key concepts underpinning a broad research area, which is particularly valuable for complex issues that have not been reviewed comprehensively to date [26-28]. In this specific context, our scoping review sought to identify articles with characteristics relevant to the uptake factors of digital therapeutics until additional articles no longer expand the argumentation and thematic saturation is reached instead of providing an exhaustive list of published literature [29]. As such, a representative sample of the digital health literature is sufficient in this context [29]. We followed Joanna Briggs Institute Manual for Evidence Synthesis for Scoping Reviews and reported the findings according to the PRISMA-ScR (Preferred Reporting Items for Systematic Reviews and Meta-Analyses extension for Scoping Reviews) guidelines (Table S1 in Multimedia Appendix 1) [30,31]. As is the case with most scoping reviews, no review protocol was published.

### Eligibility Criteria

To be eligible for inclusion, publications had to discuss factors determining digital therapeutic uptake in the health care sector. Uptake, in this context, refers to both the integration of digital therapeutics into the health system and the acceptability and practical use of digital therapeutics by patients and health professionals. Publications from 2000 onward were considered, as this year marks the start of digitalization in health [1,17,21]. Only publications in English were considered. In terms of study designs, research protocols, conference abstracts, theses, preprints, news articles, and workshop proceedings were excluded. Publications that explicitly focused on electronic health records were also excluded. Given the nature of a scoping review, the inclusion criteria were kept broad to ensure that all aspects and dimensions of the uptake factors of digital therapeutics were covered.

### Search Strategy and Data Collection

Scientific articles were systematically identified through 3 scientific databases (MEDLINE, Cochrane Database of Systematic Reviews, and Web of Science). These databases were chosen to cover both health-specific and interdisciplinary academic fields. The scientific search was supplemented with a nonsystematic search for gray literature using Google Scholar (first 200 hits) [32]. The full query for the scientific databases is shown in Table S2 in Multimedia Appendix 1. An information specialist at the London School of Economics and Political Science Library further validated the search strategy.

The initial search was conducted on December 12, 2022, and an updated search was conducted on March 9, 2023. The complete screening process (title or abstract and full-text screening) was performed by 1 reviewer in counterchronological order to ensure that recent evidence that might capture novel developments in the uptake of digital therapeutics is not overlooked. To further improve the robustness of the methodology, a subset (828/35,541, 2.33%) of the articles was also assessed by a second reviewer for verification, and interrater agreement scores were computed. Disagreements between the reviewers were resolved by consensus, and no third person was involved. The interrater agreement between the 2 reviewers was calculated using Cohen κ in R (version 4.1.2; R Foundation for Statistical Computing) [33,34]. Deduplication was performed using Endnote (version 22; Clarivate Plc), and screening was performed using Covidence (Veritas Health Innovation) [35]. Reference lists of the included articles were also screened for relevant articles.

### Data Synthesis

A thematic analysis was used to extract data relevant to the uptake of digital therapeutics [36]. Salient factors were extracted by 1 author (RvK) and clustered post hoc into relevant domains of the Consolidated Framework for Implementation Research (CFIR) [37]: (1) outer and inner settings, (2) individuals, and (3) innovation. In this review, we decided to pool the outer and inner settings into 1 domain, seeing as their contents are entangled and not mutually exclusive in the context of digital therapeutics [38]. The clustering was reviewed and verified by other authors (AR-U, MA, IK, and EM). We also applied frequency categories to the salient factors to indicate how often a particular factor was reported in the included documents: *very frequent* was applied when >15% of the included documents mention a factor, *frequent* was applied when between 5% and 15% of the included documents reported a factor, and *regular* was applied when <5% of the included documents reported a factor that can affect the uptake of digital therapeutics. It is important to note that these frequency labels do not communicate the relative importance of the uptake factors. They only capture how often a factor was mentioned in the included documents. These frequency categories were translated into color codes in the development of the map of uptake factors, with red indicating the factors very frequently cited, yellow indicating the factors frequently cited, and blue indicating the factors infrequently cited.

The results were further clustered according to Donabedian quality framework, which makes a distinction among structure, process, and outcome measures [39]. In this review, structure measures refer to the uptake factors that describe the physical, institutional, or organizational context in which care is delivered (eg, policy context or demographic characteristics). Process measures capture the transactional uptake factors of digital therapeutics (eg, the preferences, perceptions, or attitudes of patients and professionals). Outcome measures capture all the direct and indirect effects of digital therapeutics on the health care ecosystem. The policy category exclusively includes structure factors. For the patient and health professional characteristics, as well as the properties of digital therapeutics, a subclassification between structure and process measures was made. The outcome category exclusively covers outcome measures, which were divided into 4 subcategories: patient-level, health system, public health, and data science outcomes.

### Ethical Considerations

The results presented in this manuscript have no inherent ethical implications or considerations. No animals or human participants were involved in this research. No personal data were used in this research. Thus, we did not seek a review of our study design from an institutional review board.

## Results

### Search Results

**Figure 1:**
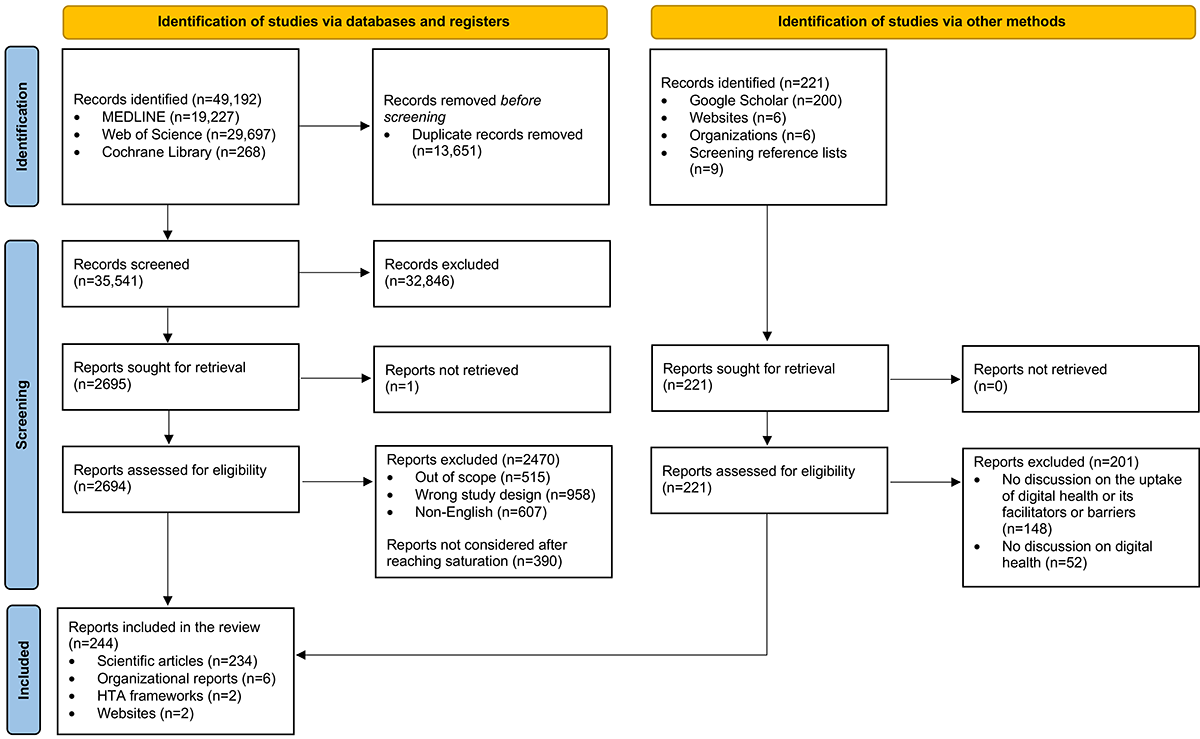
A PRISMA (Preferred Reporting Items for Systematic Reviews and Meta-Analyses) flowchart outlining the data collection process. HTA: health technology assessment.

Our search strategy yielded 49,192 results from academic database searches (n=35,541, 72.24% after deduplication) and 221 results from supplementary searches, amassing 35,762 unique records. Ultimately, out of 35,541 documents, we included 244 (0.69%) documents (n=234, 95.9% scientific articles; n=6, 2.5% organizational reports; n=2, 0.8% health technology assessment frameworks [although the included literature covered additional frameworks]; and n=2, 0.8% web pages) in this review (Figure 1). Out of 244 documents, our final sample comprised 60 (24.6%) literature reviews [19,40-98]; 49 (20.1%) viewpoints, commentaries, and editorials [11,13,17,99-144]; 43 (17.6%) cross-sectional studies [83,145-186]; 39 (16%) qualitative research articles [1,15,187-223]; 13 (5.3%) case reports [4,5,12,224-233]; 11 (4.5%) longitudinal studies [234-244]; 9 (3.7%) mixed methods studies [245-253]; 5 (2%) economic evaluations [254-258]; 5 (2%) randomized controlled trials [259-263]; 4 (1.6%) policy documents [264-267]; 3 (1.2%) preference studies [268-270]; 2 (0.8%) websites [271,272]; and 1 (0.4%) book chapter [273]. Of the 244 included studies, 23 (9.4%) were published before 2014 [41,66,71,96,98,116,122,124-126,129,130,132,158,163, 167,171,192,211,227,237,238,268]. The included articles covered 35 distinct countries from all continents. Articles representing the United States (67/244, 27.5%) constituted the largest share of the included articles, followed by those with no specific country focus (62/244, 25.4%) and those focusing on Germany (21/244, 8.6%). Figures S1 and S2 in Multimedia Appendix 1 show an overview of the article types published per geographic region. In the subset screening of the total sample by the second reviewer (828/35,541, 2.33%), we found a crude interrater agreement score of 88.6% (734/828 observations) between the 2 reviewers. We also accounted for the possibility of reaching interrater agreement by chance by computing Cohen κ (0.589), which indicated a moderate agreement between the observers.

### Outer and Inner Setting Domains

An important uptake factor of digital therapeutics was the presence of a legal framework to (1) distinguish digital therapeutics from commercial lifestyle and well-being apps, accrediting the former as a reimbursable form of health care, and (2) protect patients’ privacy, confidentiality, and (health) data security [1,4,5,13,15,47,52,54-56,59,66,77,80,87,96, 99,108,109,112,116,120,123,133,139,148,149,157,161,166,167, 169,186,190,208,209,212,231]. A clear example of such a legal framework is the mHealthBelgium validation pyramid in Belgium, which consists of 3 levels: the first level determines the basic requirements, such as the need for *Conformité Européene* marking, which indicates that a product adheres to the European Union health, safety, and environmental protection standards; the second level is designed to ensure interoperability and connectivity with the broader health informatics system; and the third level regulates the funding of digital health apps by the National Institute of Health and Disability Insurance, which is acquired after a digital health app has passed the clinical review process [224]. In terms of evidence requirements, establishing different evidence tiers to distinguish digital therapeutics from other types of digital health applications (eg, those aimed at communication or administrative functions) was considered vital [5,12,93,123,157,161,203,212,224,231,265]. In practice, this is already observed in the United Kingdom, where the National Institute of Health and Care Excellence (NICE) has devised a 3-level evidence framework that distinguishes (1) digital tools with no patient-relevant outcomes, (2) digital tools that aid in communication and education, and (3) digital therapeutics that are designed to generate and deliver medical interventions [5,224,265]. These 3 evidence tiers also each have their respective evidence requirements (Table S3 in Multimedia Appendix 1), with the highest level requiring evidence of effectiveness for the claimed benefits of the digital therapeutic on top of the requirements for the second evidence tier. The establishment of a formal body to transparently assess and certify digital therapeutics also influenced uptake [15,121,166,174,213]. Practical examples include the NICE in the United Kingdom, the Federal Institute for Drugs and Medical Devices (*Bundesinstitut für Arzneimittel und Medizinprodukte*) in Germany, and the Finnish Coordinating Center for Health Technology Assessment [75,224,229,265]. The NICE has also established an early value assessment process for digital therapeutics, which can provide preliminary insights into how digital therapeutics can benefit the health system (particularly surrounding their ability to address unmet health needs) while also assessing what types of evidence are available and what types of evidence are missing [272].

The financing model for digital therapeutics was considered highly influential with respect to their uptake in the health care system [12,65,81,87,127,139,162,170,203,205,212,229, 230,232]. In the included literature, eleven distinct financing models for digital therapeutics were identified: (1) periodical subscription (Germany and the United Kingdom), (2) 1-time license fee (Germany), (3) part of bundle packages with other (nondigital) therapeutics (the United States), (4) financing through (innovation) grants, subsidies, or fundraisers (the Netherlands and Germany), (5) sponsor-based agreements in which the digital therapeutic is sponsored by a large platform or institution (eg, Alzheimer Netherlands sponsors a digital health intervention for Dutch patients with dementia at no charge to caregivers), (6) public-private partnerships (eg, a private actor helps develop the local digital health infrastructure on the condition that their digital therapeutic is added in the local health care inventory), (7) inclusion in data plans, (8) performance-based contracts (the United States), (9) risk-benefit sharing contracts (the United States), (10) part of employment benefit plans, and (11) pay-as-you-go arrangements [12,65,86,87,127,143,162,170,203,205,212,229,230,232,239]. In addition, some form of reimbursement parity (eg, service or payment parity) with traditional biomedical therapeutics was considered important [68,70,119,123,153,169,206,238]. Incentives for the uptake of digital therapeutics were also identified as an important means of bolstering the development of a reimbursement pathway for digital therapeutics (eg, dedicated funding or financial compensation for offsetting the high fixed costs of implementing digital therapeutics in practice) [1,19,62,87,122,126,133,140,143,145,169,174,187,188,200,218,225, 227,231]. An important aspect of these incentives is the development of reimbursement codes for digital therapeutics [2,53,99,148,153,169,170,212]. The United States created reimbursement codes for Medicare and Medicaid to be able to better reimburse digital health, but these payment codes are used for digital health as a whole and not specifically for digital therapeutics [53,160]. After certification, the uptake of digital therapeutics is facilitated by the ability of health care professionals or health insurers to identify accredited digital therapeutics through an openly accessible register or inventory [4,59,224,226,229,231,232]. These registers have already been established in Germany, Belgium, and France [15,224], whereas the National Health Service (NHS) App Library in England was decommissioned in December 2021 during a restructuring of the NHS website [271].

Another vital element is the development of funding options for digital therapeutics during and after the research phase (eg, innovation and research grants, pay-for-performance constructs, or payment/completed digital therapeutic use cycle). For the latter, it is important that the financial responsibility is not placed on patients to protect them from disproportionate out-of-pocket payments and ensure that digital therapeutics do not become an exclusive domain of higher socioeconomic groups [12,15,46,58,61,72,86,99,100,105,106,114,116,146,149,154, 164,172,190,192,197,200,202,226-228,249]. It was emphasized that there were benefits to allowing the procurement of digital therapeutics as locally as possible, as health purchasers (eg, Integrated Care Boards in the United Kingdom, insurance companies in the Netherlands, and employer groups in the United States) are best placed to assess the needs and preferences of their covered population, although this could translate into longer adoption times and higher uptake costs, as each individual insurer needs to invest in the digital infrastructure. There was a stated need to adjust or redesign the existing protocols and professional guidelines, commonly created for the provision of face-to-face health care, to create opportunities for digital therapeutics and address the liability and risk-sharing concerns with using digital therapeutics [19,51,52,57,63-66,81,87,106,110,112,114,118,121,126, 139,149,158,192,205,207,225,233]. The presence or development of a high-quality digital infrastructure that can be accessed by all population groups [1,5, 13,40,45,47,63,66,74,87,100,106,119,122,124, 134,135,137,140,153,162,199,201,209,218,238,270,273], the availability of testing environments so that health professionals can gain experience with digital therapeutics [129,201], and the inclusion of digital therapeutic modules in medical curricula were identified as facilitators of the uptake of digital therapeutics [52,58,61,62,70,78,80,81,87,105,110,115,134,149,190,197, 207,208]. A good practice example of this can be found in the United Kingdom with the establishment of the NHS Digital Academy, which trains health professionals according to a comprehensive framework on digital health competencies (Figure S3 in Multimedia Appendix 1) [97,267]. Finally, the included articles emphasized the importance of developing a formal license for health professionals to allow them to prescribe digital therapeutics [13,64,68,70,87,123,124,131].

### Individuals Domain

#### Patients

In terms of the Donabedian framework structure measures, the characteristics of the population group targeted by a digital therapeutic are important uptake determinants, as they influence the impact that any digital therapeutic might have on different clinical pathways and different diseases. Demographic characteristics are associated with patients’ uptake of digital therapeutics. Younger age [5,19,40,43,49,52-54,63, 83,100,101,126,135,139,175,177,181,182,196,203,206,235,236,256,259], sex (male or female depending on the circumstances) [17,53,62,63,101,181,182,235,256], higher language skills [19,53,126], higher education level [5,17, 43,49,54,62,63,83,100,101,107,135,175,177,181,182], higher health literacy [40,47], being employed [5,17,62,100,101], higher income level [17,40,52,54,62,100,101,107, 152,153,171,175,269,270], and living in an urban environment (compared with living in a rural environment or being homeless) [17,40,53,54,62,100,101,139,156,170,171,181,258,269] were associated with increased uptake of digital therapeutics. Furthermore, cultural and social environments [49,62,71,82,86,115,135,142,159,193,195,235]; ethnic background [40,101,102,107,142,175,193,195,235]; being unhealthy or living with a disability [11,17,40,52,62,80,100-103,107,181,190,206,236,259,270]; patient-specific needs, attitudes, and habits [1,80,102,109,110,193,195]; distance to health services [152,171,185,236], risk of contracting a disease [165]; insurance status [175,270]; time since diagnosis [177]; and past health care experiences [217] can influence the uptake of digital therapeutics. The extent to which the target group can access the internet is an additional determinant of the uptake of digital therapeutics [17,19,52,53,62,63,76,77,80,88,100-102,115,137, 139,182,206,269], as well as the target group’s (perceived) digital skills and capabilities and ability to understand the risks associated with using digital tools [1,5, 13,17,40,45,49,50,52,62,63,80-82,100-102,105,107,115,135,139,147,175,177, 188,191,192,194,199-201,210,224,225,237,245,252].

For process measures, it was considered vital to understand how patients perceive the need to use the digital therapeutic (on its own as well as compared with in-person visits); their views, doubts, and comfort regarding the use of the digital therapeutic and its potential benefits; resilience to setbacks; waiting times for the health care in question; and the ease of using the digital therapeutic [13,15,19,42-44,48,50,52,62,82,83,88,107,133,141, 150,159,170,177,178,186,188,189,193,201-203,208,216, 220,221,245,247,251,252,254,269,270]. Articles from the United States and Germany indicate that individuals who rate their health status as being lower favor the use of digital tools to aid them in their health care [43,50,195], although none of these articles focused on digital therapeutics specifically. Nevertheless, patients’ preferences for using digital therapeutics instead or alongside of traditional care form a core part of whether digital therapeutics are used in practice [42,50,62,73,82,165,199,209,246,247].

#### Health Professionals

As with the target patient group, health professionals’ perceptions are also key factors of the uptake of digital therapeutics. Relevant factors include how easy the digital therapeutic is to use [13,42,44,202,218], how health professionals perceive the digital capabilities and risk profile of the patient and the quality of the digital therapeutic [90,145,172,184,211,218], how the technology will affect organizational workflow, and professional responsibilities and autonomy [13,42,54,62,110,116,133,155,189,197,198, 202,207,211,221,273]. Barriers to implementation (eg, financial, human resource, or time- and workload-related barriers) were also frequently cited [42,43,62, 63,90,104,115,139,145,155,156,199,200,202,218].

With respect to structure measures, the demographic characteristics of health professionals play a role. Female and younger health professionals were reported to favor the prescription of digital therapeutics in Germany and the Netherlands [147,196], as did health professionals located in rural environments in Australia, the United States, and Taiwan [62,151,155,236]. The digital connectivity and literacy of health professionals were widely recognized as key components in determining their willingness toward and likelihood of prescribing digital therapeutics across countries [1,5,17,42-46,62,70,76,80,81,90,100,101,104,106,116,117,135, 145,146,162,174,183,184,187,189,191,192,194,196,197,199-201,203, 210,224,225,228,231], as were their personal attitudes toward, familiarity with, and trust in digital therapeutics [1,42,43,62,70,104,105,116,139,145-147,183,184, 187,189,192,196,198-200,203,209,210,230,231,236,248].

With respect to process measures, professionals reported rigidity in their work process [146,187,192,196], as well as a tendency to be risk averse and to resist change unless a digital therapeutic had been nationally accredited or endorsed [63,104,139,147,231,248], reemphasizing the need for policy mechanisms aimed at the accreditation of digital therapeutics. Approval from the institutional or social environment was also reported as an important indicator of the uptake and use of digital therapeutics by health professionals [19,42,62,104,112,115,139,156,159,174,200,202,203]. Finally, certain medical specialties (eg, psychology, psychiatry, and neurology) may be more amenable to adopting digital therapeutics than other specialties (eg, ophthalmology, dermatology, and surgery) [140,145,146, 162,167,183,196,213,235,236,247], which may be explained by the varying degrees of availability or applicability of digital therapeutics for different medical specialties [183,196].

### Innovation Domain

#### Characteristics of Digital Therapeutics and Manufacturer Provisions

On the basis of the mapped literature, the nature, content, and structure of digital therapeutics are major determinants of their uptake and effectiveness across countries [1,4,13,19,40,63,100,102,189,191-194,200,205,230,246,265,266]. The design of digital therapeutics needs to be driven by the needs and expectations of patients [56,61,66, 68,84,100,102,117,118,122,132,136,140,141,168,177,189, 191-194,200,202,208,214-216,221,230,233,250,266,273,274] while acknowledging that patient needs may shift with time and disease progression [140]. They need to have clear aims and a position in the health care pathway that is understood by patients and professionals, with comprehensive and trusted information provided about the qualities and services of the devices and targeted health condition [56,57,93,118,142, 174,177,178,186,190,214,221,228,250,252,259, 265,266,273]. They should also be easy and straightforward to understand and use [1,4,13,19,40,42,117,135,141,150,193,194,203,208,217,250, 252,265,266,273], as well as have the ability to be personalized according to patient needs and preferences [4,13,62,117,208,216,217,220,246]. Finally, a key property and enabler of the reimbursement of digital therapeutics is their interaction with the target population: they should be co-designed and coimplemented with patients and health professionals to maximize the likelihood of their uptake by the relevant stakeholders, with the recognition of the fact that engagement might differ across population groups in different countries [4,13,19,40,47,56,57,89,129,166,174,189, 190,200,214,215,220,230,250].

With respect to structure measures, the interoperability of digital therapeutics (ie, the ability to communicate with local health informatics systems, such as electronic health records) was the most often emphasized requirement from a design viewpoint [1,4,13,15,17,19,44,45,54,57,61,66,80,82,103,106,107, 110,111,124,147,189-192,197,199,200,202,222,224, 230,231,246,264-266,270,273]. However, it is important that digital therapeutics remain compliant with local privacy and security legislation and prevent direct-to-consumer advertisements through the application [4,47-49,51,57,93,115,125,138,187,211,219,220,224], as well as identification through secondary data sharing [79,91,93,138,217]. The hardware and software requirements [63,91,163,194,233], implementation and upkeep costs [41,63,65,133,146,198,211,231,237,238,254], required input and oversight from health professionals [11,107,254,265], ability to work without a constant internet connection [91,135,217,233], price [165,222], and scalability were also deemed important factors, as they dictate the investment threshold for adopting digital therapeutics and how widely and cost-effectively digital therapeutics can be deployed, including the potential to realize economies of scale [164,199,231,265].

With respect to process measures, certain additional provisions from the developer broaden the potential uptake of digital therapeutics in addition to enhancing the digital therapeutics themselves. These additional provisions include dedicated training courses for the digital therapeutic aimed at patients and health professionals, and user support during and after implementation were noted as critical uptake factors [13,19,61-63,66,73,115,118,145,148,159,184,199,203,206,252].

Finally, a key property of digital therapeutics was the existence of a strong evidence base that matches the corresponding evidence tier of the national regulatory framework. This should contain data on the positive health effects of using the digital therapeutic compared with the status quo or preexisting treatment options (eg, pragmatic randomized controlled trials using a real-world data arm), the context in which the digital therapeutic is to be implemented, how it affects the clinical workflow in the target setting, economic evaluations (cost-effectiveness, cost utility, and cost benefit), economic risk to the payer, budget impact, qualitative evidence on patients’ and health professionals’ experiences with using the tool, health system needs assessment, the safety of the patient and their data (eg, reports of regular penetration testing), and transferability and generalizability across population groups [1,4, 13,15,44,46,49,54,61,65,85,86,91,98,105,106,108-112,117, 118,133,144,188,196,197,200,210,228,229,232,266]. In this context, positive health effects include not only medical benefits, such as improved health status, shortening of the duration of disease, prolongation of survival, and improvement of the quality of life, but also broader elements, such as treatment adherence, patient safety and sovereignty, coping capabilities, and the reduction of therapy-related efforts [108,109,229].

#### Relative Advantages of Digital Therapeutics

The mapped literature highlighted the multidimensionality and versatility of digital therapeutics by showcasing their relative advantages compared with their competitors. At the patient level, the most reported relative advantages were improvements in health outcomes and shortened disease duration through novel health care options, faster diagnoses, decrease in adverse medical events, and improved monitoring of clinical and patient-reported outcome measures [11,17,41,43, 45,47-49,53,60,61,69,71,74,80,81,88,92,94,95,100,102,105,124,128,130, 132,133,135,137,138,146,153,163,172,173,179,189,202,207,218,224,226,228, 229,231,232,234,239-242,244,246,248,257,260,262,263,265,266,270,275], as well as changes in the quality of life, well-being, and life expectancy [13,47,48,58,88,92,120,128,153,224,226, 229,244,246,257]. Digital therapeutics can also induce changes in the quality of care, patient satisfaction, and diagnostic accuracy [48,52,53,55,60,69,76,88,89,92,102,128,137, 147,165,168,179,189,203,219,242,243,270,273] while empowering the patient to have a more active role in their health care and further enabling shared decision-making [4,56,82,92,102,105,134,139,175,189,191,201, 216,225,226,229,231,241,246,248,258,266,273]. At the behavioral level, digital therapeutics have been shown to positively affect treatment adherence [41,48,69,137,196,226,229,231,246,266,273], patients’ coping and health management abilities [4,13,76,82,126,134,137,145,175,226,229,244,254,257,263,266,270,273], and general behavioral and lifestyle aspects [13,43,56,246,254]. Furthermore, digital therapeutics could improve access to health care, reduce waiting times, and lower thresholds for seeking out health services [1,41,43,45,48,49,53,55,61,62,67,70,71, 74,76,80,88,90,91,95,99,102,111,113,120,124,130-133,137-139,142,150, 151,162,171,173,181,183,185,193,196,203,216,217,224-226,229,232,234, 239,240,242,247,249,253,256,261,262,269,273,275]. They can also aid in the development of (digital) health literacy [4,13,41,43,47,76,82,83,89,92,139,147,165,196,202,208, 219,226,229,242,250,251,257,266,270,274].

At the health system level, digital therapeutics can influence health care costs and resource use [41,47,48, 53,69,71,72,80,81,94,95,105,107,111,113,121,124,128,131,133,147, 153,154,160,164,171,172,180,189,207,211,212,228,231,232,237,238,255-258, 261,262,265,269,273] and affect the workload and workflow of the health workforce, potentially bringing about significant improvements [19,47,60,62,78,80,90,105,116,124,126,128, 134,146,147,176,188,194,196,221,231]. They can also improve coordination among health professionals [60,90,92,225,226,229,232,273]. Furthermore, as they require less health professional input, digital therapeutics can help address clinical workforce shortages [47,72,80,124,128,173,176,228,243,270].

Digital therapeutics have secondary relative advantages at the public health and data science levels. The data generated through their use can allow for the tracking of disease burden [1,100,151,253], resulting in the ability to monitor and model the spread patterns of infectious diseases and inform national priorities for health care procurement and resource allocation [1,17,41,45,151]. As they reduce the need for patients to travel to health care facilities, they are both environmentally friendly and can reduce the risk of hospital-borne infections [120,139,165,171,180,185]. When designed and deployed adequately, digital therapeutics can also contribute to the reduction of health inequalities; however, when designed poorly or without the consideration of the potential for the digital exclusion of groups with lower levels of digital literacy, they have the potential to exacerbate health inequalities [47,53,68,70,81,95,99,100,102,107,120,127,132,139,140, 142,143,173,182,210,218,221,242]. Finally, digital therapeutics can aid in the generation and analysis of meaningful real-world (health) data through their integration with electronic health records [5,136,231,273], enable the use of cloud-based diagnostics and (big data) analyses [48,72,223], and be continuously developed and improved through iterative user feedback [201]. However, privacy-conscious data-sharing platforms (eg, PhysioNet and the US National Institute of Health *All of Us* research program) are vital for minimizing the risk of reidentification [79].

### Comprehensive Map of Uptake Factors

**Figure 2:**
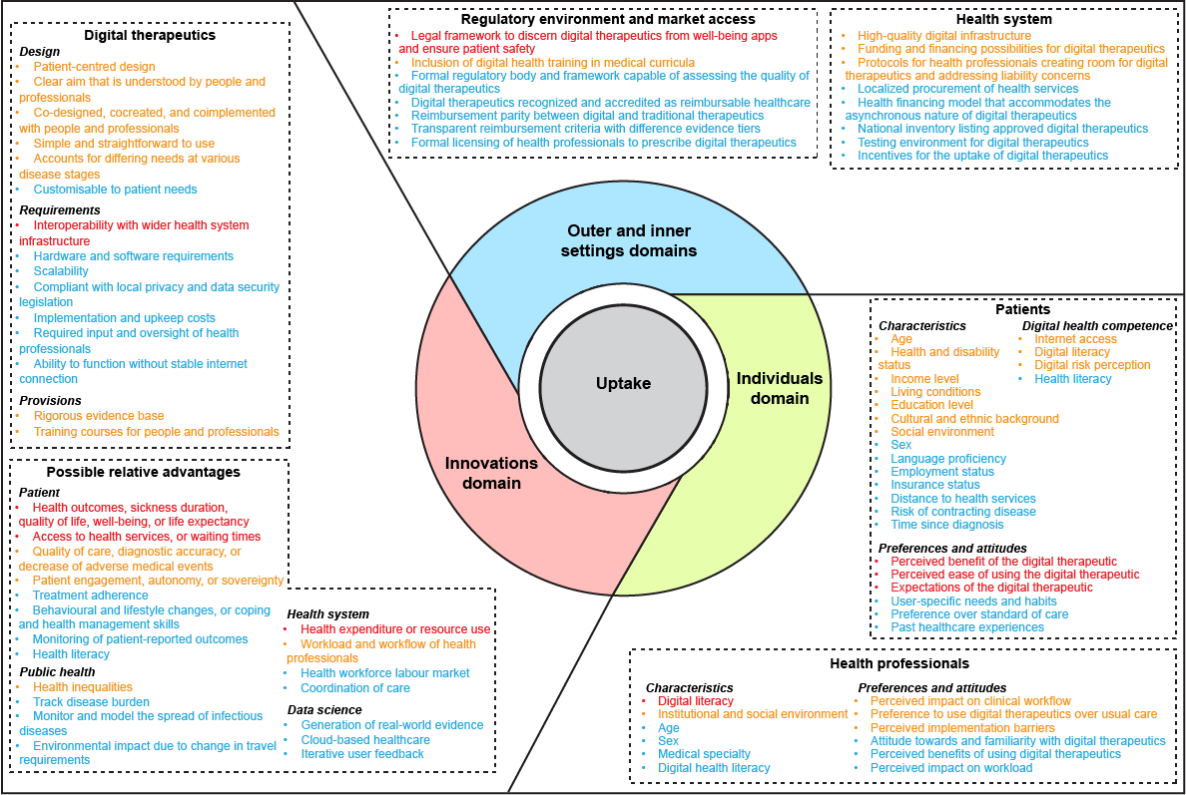
Map of the factors that can affect the uptake of digital therapeutics. The items in red are reported in 15% or more of the included articles. Items in yellow are reported in 5% to 15% of included articles. Items in blue are reported in less than 5% of the included articles.

From the 244 included documents, we extracted 85 factors that determine the uptake of digital therapeutics within a health system. At the policy level, the need to develop a legal framework for digital therapeutics was very frequently iterated (37/244, 15.2%). As for patients, their demographics (57/244, 23.4%), perceptions of digital therapeutics (39/244, 16%), and digital skills and risk perceptions (38/244, 15.6%) were very frequently reported to influence the potential uptake of digital therapeutics. The interoperability of digital therapeutics was also very frequently reported to influence their uptake (39/244, 16%). As for health professionals, their digital connectivity and literacy were the most stated factors affecting their uptake of digital therapeutics (43/244, 17.6%). In terms of the relative advantages of digital therapeutics, improved access to health services (65/244, 26.6%), improved health outcomes (63/244, 25.8%), and changes in health care costs and resource use (46/244, 18.9%) were very frequently reported. Further details on the frequency with which uptake factors were reported in the included studies are shown in Table 1. Finally, a visual map that outlines all the extracted factors color coded by frequency is shown in Figure 2.

**Table 1:** Frequency table of the most reported uptake factors in the included documents (n=244).

Domain and factors				Frequency, n (%)
**Policy**				
	**Very frequently reported factors (≥15%)**			
		Developing a legal framework	37 (15.2)	
	**Frequently reported factors (5%-15%)**			
		Implementing a high-quality digital infrastructure	28 (11.5)	
		Developing funding options	27 (11.1)	
		Redesigning protocols and professional guidelines	25 (10.2)	
		Inclusion of digital health modules in medical education	18 (7.4)	
		Financing model for digital therapeutics	17 (7)	
**Patient characteristics**				
	**Very frequently reported factors (≥15%)**			
		Demographic characteristics	57 (23.4)	
		Patient perceptions of digital therapeutics	39 (16)	
		Digital skills and risk perceptions	38 (15.6)	
**Digital therapeutic**				
	**Very frequently reported factors (≥15%)**			
		Interoperability	39 (16)	
	**Frequently reported factors (5%-15%)**			
		Need-driven and patient-centered design	34 (13.9)	
		Evidence base	33 (13.5)	
		Simple and straightforward to use	20 (8.2)	
		Clear aim that is understood by patients and professionals	19 (7.8)	
		Accounting for different needs across populations and disease stages	19 (7.8)	
		Provision of training courses for patients and professionals	17 (7)	
		Remaining compliant with local privacy and security legislation	15 (6.1)	
**Health professional**				
	**Very frequently reported factors (≥15%)**			
		Digital literacy	43 (17.6)	
	**Frequently reported factors (5%-15%)**			
		Professional preference	28 (11.5)	
		Effect on clinical workflow and professional autonomy	16 (6.6)	
		Financial, human resource, or time- and workload-related barriers	15 (6.1)	
		Approval from the institutional or social environment	13 (5.3)	
**Outcome**				
	**Very frequently reported factors (≥15%)**			
		Improved access to health services	65 (26.6)	
		Improved health outcomes	63 (25.8)	
		Change in health care costs and resource use	46 (18.9)	
	**Frequently reported factors (5%-15%)**			
		Improving digital literacy, health literacy, or digital health literacy in patients	26 (10.7)	
		Improving the quality of care, patient satisfaction, and diagnostic accuracy	24 (9.8)	
		Enabling shared decision-making	23 (9.4)	
		Affecting health inequalities	23 (9.4)	
		Affecting the workload and workflow of the health workforce	21 (8.6)	

## Discussion

### Principal Findings

This review aimed to create the first map of the most frequently discussed uptake factors of digital therapeutics that will aid their implementation across different health systems. Our map contains 85 factors that can affect the uptake of digital therapeutics within a health system. This map offers a novel and comprehensive overview of the known uptake factors from both technological and human perspectives. This bridges the dichotomy of the digital health literature in which articles either focus on the technological or patient and professional perspectives, which inadvertently highlights the novelty of this review. The map is intended to be scaled and adopted by countries looking to introduce digital therapeutics in their health service portfolio, expand the proportion of their population that benefits from digital therapeutics, or extend access to digital therapeutics to excluded communities. As this map was based on a standardized set of constructs in the CFIR, it can support future research on and implementation endeavors of digital therapeutics. It offers policy makers, health insurers, and other change agents specific digital health–related action points to focus on in their pursuit of implementing digital therapeutics in the health care system. In doing so, it can help address the existing barriers to the uptake of digital therapeutics, harmonize digital therapeutic infrastructure across health systems, and aid researchers in analyzing health system readiness for using digital therapeutics.

This map of factors aims to guide the integration of digital therapeutics into clinical practice by helping stakeholders identify, assess, and address key uptake barriers unique to digital therapeutics, including issues related to policy readiness, regulatory approval, reimbursement, engagement, data privacy and security, adoption, and clinical effectiveness. It highlights the finding that the innovation and individuals domains of the CFIR contain the most frequently cited uptake factors of digital therapeutics. The relative focus on these factors is unsurprising, seeing how digital therapeutics are unprecedented in terms of the required user input. It also signals these uptake factors as action points for policy makers, health insurers, and health promotion experts. Patient groups may need to be sensitized to the concept of digital therapeutics before they are ready and willing to use these novel tools in their health care pathways [22]. Simultaneously, it points to an opportunity for investors to update funding requirements to better enfranchise future users of digital therapeutics, especially if they are designed to combat the existing health inequalities.

Users of this map should consider the unique characteristics and needs of different health care settings and digital therapeutics on a per-case basis. Consequently, health care organizations can ensure that they are able to effectively leverage the potential of digital therapeutics to improve patient outcomes and address pressing health care challenges [1,71,128,194]. The map is not solely reliant on clinical health outcomes but allows room for a broader interpretation of positive health effects, such as treatment adherence, patient safety and sovereignty, coping capabilities, and reduction of therapy-related efforts [109,229]. This interpretation allows for a more versatile and holistic assessment of digital therapeutics, which could contribute to the value-based pricing of digital therapeutics [12,229].

The focus on the United States in the digital health literature included in this review is likely related to the current health system challenges worldwide. In particular, the United States is experiencing stark rises in health care costs, inequitable access to health care, and labor shortages in the health workforce [276], for which digital therapeutics can be seen as a potential (partial) solution. Although these issues exist worldwide, the United States has a favorable environment for developing and adopting innovations, as it hosts the largest technology companies and venture capital investors [277]. By contrast, the focus on Germany, for example, is attributable to the recent adoption of the Digital Healthcare Act, which only now formally enabled digital therapeutics to be prescribed by general practitioners [266].

In this review, we also found that countries with multipayer reimbursement systems (the United States, Germany, and the Netherlands) form the predominant countries of interest in the literature on reimbursing digital therapeutics. These health systems are built on the basis of (managed) competition and a mix of supplementary and substitutive private health insurance markets [278]. Stereotypically, these types of systems are more conducive to adopting health care innovations, as insurers are actively incentivized to look for a competitive advantage and potential cost-saving solutions [279]. This reasoning also suggests a partial explanation for why countries with a single-payer system (eg, Australia, Canada, or the European Nordic countries) are poorly represented in the current digital health literature. The outlier here is the United Kingdom, where there has been a broad policy discussion around this topic with a strong impetus from national bodies (eg, NHS England) [280]. A downside of introducing digital therapeutics in multipayer countries is the high investment cost of setting up the digital infrastructure in a health system. The Netherlands offers a partial solution to this problem by allowing health insurers to pool their resources and collectively buy the digital infrastructure required to run digital therapeutics [281].

### Limitations and Future Research

Some limitations of this review need to be considered. First, the findings of this review should be interpreted as scoping, meaning it provides a high-level overview of the literature and may not capture more intricate and field-specific factors that can affect the uptake of digital therapeutics in health care. Second, the quality of the included sources was not assessed, which should be considered when interpreting the results. Third, we acknowledge the presence of evidence selection bias, as only 3 academic databases and Google Scholar were used, and the search strategy was not exhaustive. That said, an exhaustive search was not required to reach the aim of this review, namely the development of a map of uptake factors. In fact, we reached data saturation during the extraction of the current sample of articles. Fourth, we acknowledge that this review only captures the uptake factors that are considered important within the current digital health literature. Fifth, the high representation of the United States in the included articles might skew our results to be geared toward the US health ecosystem, although we believe that this is counterbalanced by the articles that focused on countries other than the United States. Sixth, these results cannot be directly transferred to the context of artificial intelligence, even though it overlaps to some degree with digital therapeutics, as the latter are often powered by some form of artificial intelligence. Finally, we acknowledge that this review makes broad conclusions about digital therapeutics holistically and may not be applicable to individual applications.

Various avenues for further research were also identified in this review. A comprehensive evidence synthesis for each domain within the map of factors could further improve its quality and robustness. An assessment of the relative importance of the uptake factors within each domain can provide more actionable guidance for policy makers, health professionals, and patients. Patient engagement and empowerment lie at the core of digital therapeutics, yet these concepts remain unquantified. Future research should investigate conceptualizing key performance indicators for patient activity and engagement with digital therapeutics. Even though qualitative studies comprised a decent proportion of the included studies (39/244, 16%), these studies focused predominantly on the experiences of implementing digital health applications in finalized or near-finalized development stages into practical situations. However, research on the process by which a digital therapeutic is developed and how patients can be enfranchised within that process is lacking. Future research should also investigate the possibility, risks, and benefits of migrating the necessary health data infrastructure for digital therapeutics to cloud-based environments compared with local storage. Finally, future research should explore how different financing models for digital therapeutics interact with different types of health systems to identify best practices and further streamline the integration of digital therapeutics in health care pathways.

### Conclusions

Ultimately, digital therapeutics are set to disrupt the delivery of health care by challenging the fundamental assumption that health care needs to be location bound and episodic in nature [130]. Understanding the array of factors that determine the uptake of digital therapeutics in the health care landscape is a vital step in progressing the development of digitally augmented health systems. The map of factors developed in this review offers a multistakeholder approach to recognizing the uptake factors of digital therapeutics in the health care pathway and provides an analytical tool for policy makers to assess their health system’s readiness for digital therapeutics.

## Appendix

Multimedia Appendix 1 Additional figures, tables, and the search strategy per database.

## Abbreviations

CFIR: Consolidated Framework for Implementation Research
NHS: National Health Service
NICE: National Institute of Health and Care Excellence
PRISMA-ScR: Preferred Reporting Items for Systematic Reviews and Meta-Analyses extension for Scoping Reviews
