# Supplementary material for: Mapping Factors That Affect the Uptake of Digital Therapeutics Within Health Systems: Scoping Review
Source: J Med Internet Res. 2023 Jul 25;25:e48000. doi: 10.2196/48000 (PMC10410406; doi:10.2196/48000)
Supplement: Multimedia Appendix 1 [file jmir_v25i1e48000_app1.docx]

**Supplementary Materials**

van Kessel, R., Roman-Urrestarazu, A., Anderson, M., Kyriopoulos, I., Field, S., Monti, G., Reed, S., Pavlova, M., Wharton, G., Mossialos, E. Mapping Factors That Affect the Uptake of Digital Therapeutics Within Health Systems: A Scoping Review

**Table S1. Preferred Reporting Items for Systematic reviews and Meta-Analyses extension for Scoping Reviews (PRISMA-ScR) Checklist**

| **SECTION** | **ITEM** | **PRISMA-ScR CHECKLIST ITEM** | **REPORTED ON PAGE #** |
| --- | --- | --- | --- |
| **TITLE** | | | |
| Title | 1 | Identify the report as a scoping review. | 1 |
| **ABSTRACT** | | | |
| Structured summary | 2 | Provide a structured summary that includes (as applicable): background, objectives, eligibility criteria, sources of evidence, charting methods, results, and conclusions that relate to the review questions and objectives. | 2 |
| **INTRODUCTION** | | | |
| Rationale | 3 | Describe the rationale for the review in the context of what is already known. Explain why the review questions/objectives lend themselves to a scoping review approach. | 3-4 |
| Objectives | 4 | Provide an explicit statement of the questions and objectives being addressed with reference to their key elements (e.g., population or participants, concepts, and context) or other relevant key elements used to conceptualize the review questions and/or objectives. | 4 |
| **METHODS** | | | |
| Protocol and registration | 5 | Indicate whether a review protocol exists; state if and where it can be accessed (e.g., a Web address); and if available, provide registration information, including the registration number. | 4 |
| Eligibility criteria | 6 | Specify characteristics of the sources of evidence used as eligibility criteria (e.g., years considered, language, and publication status), and provide a rationale. | 4 |
| Information sources* | 7 | Describe all information sources in the search (e.g., databases with dates of coverage and contact with authors to identify additional sources), as well as the date the most recent search was executed. | 4 |
| Search | 8 | Present the full electronic search strategy for at least 1 database, including any limits used, such that it could be repeated. | 4 |
| Selection of sources of evidence† | 9 | State the process for selecting sources of evidence (i.e., screening and eligibility) included in the scoping review. | 4 |
| Data charting process‡ | 10 | Describe the methods of charting data from the included sources of evidence (e.g., calibrated forms or forms that have been tested by the team before their use, and whether data charting was done independently or in duplicate) and any processes for obtaining and confirming data from investigators. | NA |
| Data items | 11 | List and define all variables for which data were sought and any assumptions and simplifications made. | NA |
| Critical appraisal of individual sources of evidence§ | 12 | If done, provide a rationale for conducting a critical appraisal of included sources of evidence; describe the methods used and how this information was used in any data synthesis (if appropriate). | NA |
| Synthesis of results | 13 | Describe the methods of handling and summarizing the data that were charted. | 5 |
| **RESULTS** | | | |
| Selection of sources of evidence | 14 | Give numbers of sources of evidence screened, assessed for eligibility, and included in the review, with reasons for exclusions at each stage, ideally using a flow diagram. | 5-6 |
| Characteristics of sources of evidence | 15 | For each source of evidence, present characteristics for which data were charted and provide the citations. | 5-6 |
| Critical appraisal within sources of evidence | 16 | If done, present data on critical appraisal of included sources of evidence (see item 12). | NA |
| Results of individual sources of evidence | 17 | For each included source of evidence, present the relevant data that were charted that relate to the review questions and objectives. | NA |
| Synthesis of results | 18 | Summarize and/or present the charting results as they relate to the review questions and objectives. | 8-13 |
| **DISCUSSION** | | | |
| Summary of evidence | 19 | Summarize the main results (including an overview of concepts, themes, and types of evidence available), link to the review questions and objectives, and consider the relevance to key groups. | 13-15 |
| Limitations | 20 | Discuss the limitations of the scoping review process. | 16 |
| Conclusions | 21 | Provide a general interpretation of the results with respect to the review questions and objectives, as well as potential implications and/or next steps. | 16 |
| **FUNDING** | | | |
| Funding | 22 | Describe sources of funding for the included sources of evidence, as well as sources of funding for the scoping review. Describe the role of the funders of the scoping review. | 17 |

**Table S2. Differentiation of the search string per database or search engine.**

| **Database** | **Query** | **Hits** |
| --- | --- | --- |
| **Medline (Ovid)** | *1 (digital adj3 (health or medicine or therapeutic? or care)).ti,ab.*  *2 (ehealth or e-health or mhealth or m-health or telehealth or telemedicine or "health app*" or telecare or "virtual health" OR "mobile app*").ti,ab.*  *3 exp telemedicine/*  *4 1 or 2 or 3*  *5 Electronic Health Records/ or Medical Records Systems, Computerized/ or Medical Records/ or hospital records/ or nursing records/*  *6 ("electronic patient record*" or "electronic medical record*" or "electronic health record*" or "computeri#ed patient record*" or "computeri#ed medical record*" or "computeri#ed health record*" or "ambulatory medical record*" or EHR or AMR or EPR or "patient record*" or "medical record*" or "health record*").ti,ab.*  *7 exp Medical Records Systems, Computerized/*  *8 5 or 6 or 7*  *9 4 not 8*  *10 (value or benefit* or utility or cost-effective* or pric* or financ* or reimburs* or insurance or cover* or purchas* or inequalit* or equit*).ti,ab.*  *11 9 and 10* | 19227 |
| **Web of Science** | *(TS=(digital NEAR/3 (health or medicine or therapeutic? or care)) OR TS=(ehealth or e-health or mhealth or m-health or telehealth or telemedicine or "health app*" or telecare or "virtual health" OR "mobile app*”) NOT TS=("electronic patient record*" or "electronic medical record*" or "electronic health record*" or "computeri?ed patient record*" or "computeri?ed medical record*" or "computeri?ed health record*" or "ambulatory medical record*" or EHR or AMR or EPR or "patient record*" or "medical record*" or "health record*")) AND TS=(value or benefit* or utility or cost-effective* or pric* or financ* or reimburs* or insurance or cover* or purchas* or inequalit* or equit*)* | 29697 |
| **Cochrane** | *#1 MeSH descriptor: [Telemedicine] explode all trees*  *#2 "digital health" or "digital medicine" or "digital therapeutic*" or ehealth or e-health or mhealth or m-health or telehealth or telemedicine or "health app*" or telecare or "virtual health" OR "mobile app*"*  *#3 #1 or #2*  *#4 "electronic patient record*" or "electronic medical record*" or "electronic health record*" or "computeri#ed patient record*" or "computeri#ed medical record*" or "computeri#ed health record*" or "ambulatory medical record*" or EHR or AMR or EPR or "patient record*" or "medical record*" or "health record*"*  *#5 #3 not #4*  *#6 value or benefit* or utility or cost-effective* or pric* or financ* or reimburs* or insurance or cover* or purchas* or inequalit* or equit**  *#7 #5 and #6 with Cochrane Library publication date Between Jan 2000 and Mar 2023, in Cochrane Reviews* | 268 |

**Figure S1. Distribution of all articles per article type per included geographical region.**

**Figure S2. Distribution of peer-reviewed articles per article type per included geographical region.**

**Table S3. An overview of the different evidence requirements between evidence tiers in the NICE evidence framework.**

| **Evidence standard** | **Evidence tier** | | |
| --- | --- | --- | --- |
| **Design factors** | **Tier 1** | **Tier 2** | **Tier 3** |
| The digital health technology (DHT) should comply with relevant safety and quality standards | X | X | X |
| Incorporate intended user group acceptability in the design of the DHT | X | X | X |
| Consider environmental sustainability | X | X | X |
| Consider health and care inequalities and bias mitigation | X | X | X |
| Embed good data practices in the design of the DHT | X | X | X |
| Define the level of professional oversight | X | X | X |
| Show processes for creating reliable health information |  | X | X |
| Show that the DHT is credible with UK professionals |  | X | X |
| Provide safeguarding assurances for DHTs where users are considered to be in vulnerable groups, or where peer-to-peer interaction is enabled |  | X | X |
| **Describe value** |  |  |  |
| Describe the intended purpose and target population | X | X | X |
| Describe the current pathway or system process | X | X | X |
| Describe the proposed pathway or system process using the DHT | X | X | X |
| Describe the expected health, cost and resource impacts compared with current care or system processes | X | X | X |
| **Demonstrating performance** |  |  |  |
| Provide evidence of the DHT's effectiveness to support its claimed benefits |  |  | X |
| Show real-world evidence that the claimed benefits can be realised in practice | X | X | X |
| The company and evaluator should agree a plan for measuring usage and changes in the DHT's performance over time | X | X | X |
| **Delivering value** |  |  |  |
| Provide a budget impact analysis | X | X | X |
| For DHTs with higher financial risk, provide a cost-effectiveness analysis | X | X | X |
| **Deployment considerations** |  |  |  |
| Ensure transparency about requirements for deployment | X | X | X |
| Describe strategies for communication, consent and training processes to allow the DHT to be understood | X | X | X |
| Ensure appropriate scalability | X | X | X |
| Note: Evidence standards have been copied verbatim from the evidence framework developed by NICE | |  |  |

**Figure S3. Artificial Intelligence (AI) and Digital Healthcare Technologies Capability framework. Adapted from NHS Health Education England.**^1^ **Visual is the authors' own creation.**

**
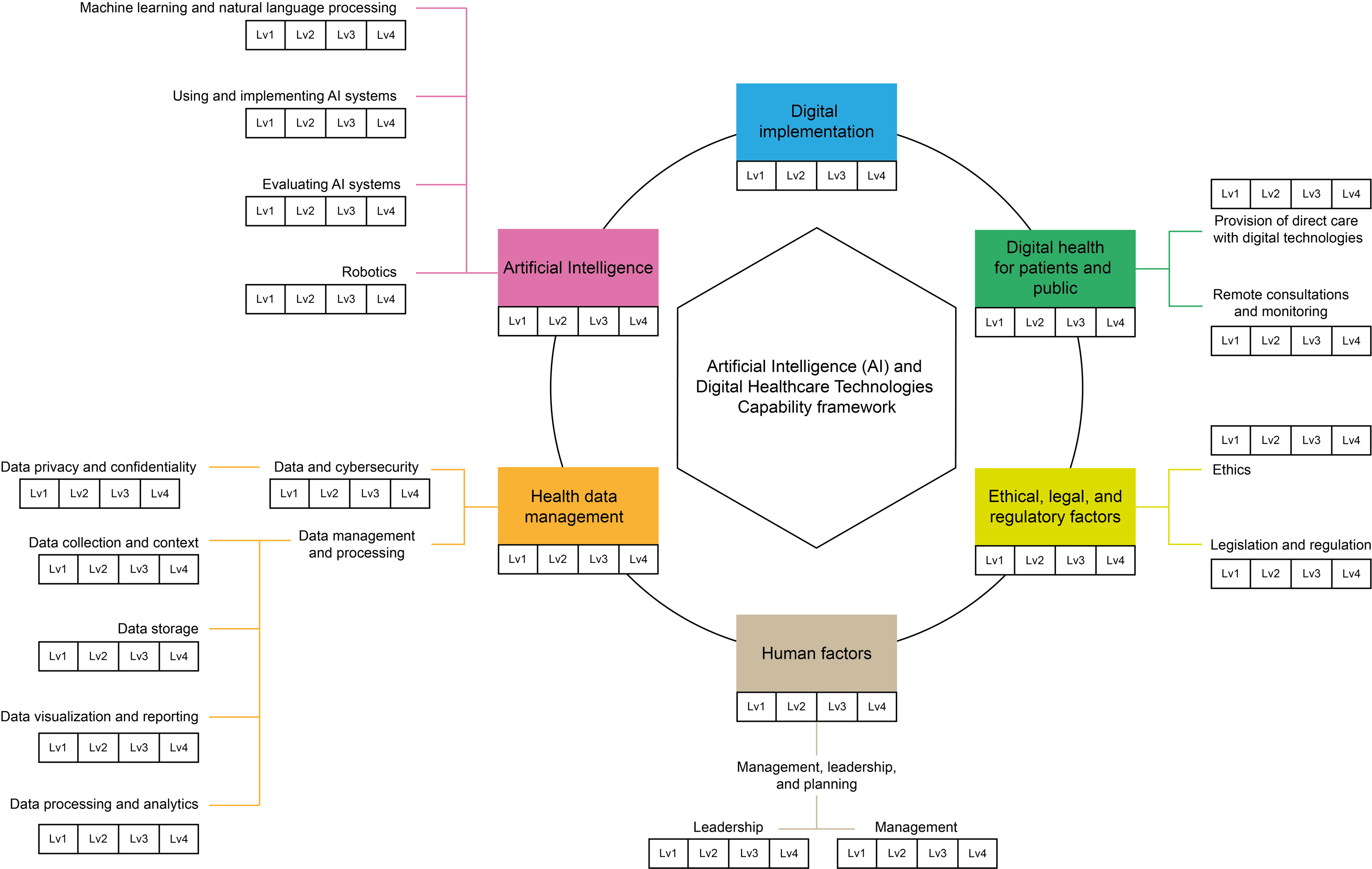
**

**Supplementary References**

1. NHS Health Education England. Artificial Intelligence (AI) and Digital Healthcare Technologies Capability framework. *NHS Health Education England: Digital Transformations* https://digital-transformation.hee.nhs.uk/building-a-digital-workforce/dart-ed/horizon-scanning/ai-and-digital-healthcare-technologies (2023).
